# Affectivism about intuitions

**DOI:** 10.1007/s11229-022-03749-0

**Published:** 2022-06-25

**Authors:** Slawa Loev

**Affiliations:** Cognition, Values, Behaviour Lab (CVBE), Ludwig-Maximilians-Universität München, Gabelsbergerstr. 62, 80333 Munich, Germany

**Keywords:** Intuitions, Emotional experience, Emotion, Epistemic feelings, Phenomenology, Justification, Reductionism

## Abstract

This article provides an account of intuitions: Affectivism. Affectivism states that intuitions are emotional experiences. The article proceeds as follows: first, the features that intuitions are typically taken to have are introduced. Then some issues with extant theories are outlined. After that, emotional experiences and their central features are brought into view. This is followed by a comparison of intuitions and emotional experiences, yielding the result that emotional experiences fit and elucidate the feature profile of intuitions. Finally, it is specified what kind of emotional experiences intuitions are: intuitions are typically mild emotional experiences that belong to the subclass of epistemic feelings.

## Introduction

There is exactly one line between any two points.

1729 is the smallest number expressible as the sum of two positive cubes in two different ways.^1^

You might have an intuition that the first proposition is true and no such intuition about the second proposition. What are intuitions? Here I answer this question by substantiating Blaise Pascal’s insight that intuitions are creatures of “the heart”. I put forward the thesis that intuitions are emotional experiences.

Why do we need another account of intuitions? This is because the current choice is an unhappy one and the dialectic surrounding it in a stalemate. Let me explain. Concerning the choice, there are two traditional flavours to pick from: Reductionism and Non-Reductionism. Reductionism states that intuitions are some familiar kind of mental state while Non-Reductionism takes intuitions to be irreducible to other kinds of mental states. So far there is one brand of each on the market: Doxasticism and Perceptualism, respectively.^2^ Doxasticism reduces intuitions to doxastic states such as beliefs and judgments. Perceptualism maintains that intuitions are a *sui generis* kind of state that bears some resemblance to perceptual experience (while remaining distinct from it). Specifically, Perceptualists describe intuitions as non-doxastic states with a characteristic phenomenology. This phenomenology might come to the fore if you consider propositions such as:

If p, then not-not-p.^3^ (Non-Contradiction)

Physical objects continue to exist when we do not perceive them.^4^ (Permanence)

A cylinder with a certain base and height encloses a greater volume than a cone with the same base and height.^5^

The idea is that engagement with such propositions might prompt intuitions and that there is a special way “it is like” to have such.^6^ A common way to refer to this phenomenology is to say that a proposition “seems” or “appears true”.^7^ Doxasticism and Perceptualism have significant qualities: On the one hand, Doxasticism is parsimonious and secures a stable place for intuitions in our mental ontology. Perceptualism, on the other hand, acknowledges that intuitions have a characteristic phenomenology and can play a non-inferential justificatory role akin to perceptual experiences.

The unhappiness of the choice comes in the form of a dilemma when having to decide between the two: Going for Doxasticism significantly deflates the phenomenology and justificatory force of intuitions while opting for Perceptualism comes at the cost of an inflationary ontology.^8^ Thus, intuitions either come with a robust ontology or else a substantial phenomenology and epistemology—but not with all three.

A consequence of this dilemma is a dialectical stalemate between the two camps. A major point of contention is phenomenology: Doxasticists dismiss Perceptualism based on a reported absence of intuitive phenomenology while Perceptualists dismiss Doxasticism based on a reported presence of intuitive phenomenology.^9^ So far Doxasticists have been unable to offer Perceptualists a reductionist analysis attractive enough in phenomenal and epistemic terms while Perceptualists have been unable to sway Doxasticists with appeals to intuitive phenomenology carried by *sui generis* posits. The result is a stalemate that is unlikely to be resolved by appeals to phenomenology.

In this article I outline a reductionist and non-doxastic account of intuitions: Affectivism about intuitions. Affectivism states that intuitions are emotional experiences (or “emotions” for short).^10^ Emotions have a firm place in our mental ontology, a canonical phenomenology and might play epistemic roles akin to perceptual experiences. Affectivism about intuitions thus promises an appealing bundle of ontology, phenomenology and epistemology. As such, Affectivism might be able to resolve the stalemate by substituting appeals to phenomenology with a reductionist metaphysical claim.^11^

I will proceed as follows: first, I introduce features that intuitions are typically taken to have. I then outline some issues with extant theories of intuitions. Then I bring emotional experiences into view and sketch some of their central features. I go on to compare intuitions and emotional experiences, pointing out that emotional experiences fit and elucidate the feature profile of intuitions. I then make some more specific claims about what kinds of emotional experiences intuitions are: typically mild emotional experiences that belong to the subclass of epistemic feelings.

## Features of intuitions

What makes a theory of intuitions a good theory? A good theory should acknowledge and explain the features of intuitions. I will present a selection of these features. Call it the “feature profile of intuitions”. Why these features? First, one finds these features in the intuition literature. I am persuaded by this literature and find the mentioned features in my own experience (and hope that you do, too). Second, I think that together they constitute a cluster of properties that will capture *only* instances of the target state, although perhaps not all of them.^12^

What is it like to have an intuition about propositions such as *Non-Contradiction*? A phenomenal contrast might help. Consider for instance:

The negation of a disjunction is the conjunction of the negations.^13^ (De Morgan’s Law)

If you have never considered *De Morgan’s Law* you might not instantly have the intuition that it is true. But after some reflection, something might happen: Suddenly, it just starts to seem true to you (Bealer, 1992). This is a modification in your phenomenal state brought about by the occurrence of an intuition. Something similar is the case for the phenomenal contrast between considering *2* + *2* = *4* and *6253* + *4773* = *11,026* or the above-mentioned propositions and propositions such as:

If p, then not not-not-not-not-not-not-not-p.^14^

A cylinder with a certain base and height encloses three times the volume as a cone with the same base and height.^15^

This is not to say that one cannot have intuitions concerning these propositions. But without special preconditions (e.g. giftedness or expertise) these propositions are less likely than the ones above to elicit intuitions.

Now, what does this contrast consist in? What properties are present when you have an intuition and are absent when you don’t?

First, intuitions are INTENTIONAL states, they are *about* something, say, about propositions such as *Non-Contradiction* (e.g. Koksvik, 2021; Sosa, 2007).^16^ These propositions are the intentional objects of the intuitions and the intuitions can be said to be about or to represent their intentional objects, i.e. the specific propositions.^17^

Furthermore, an intuition represents its content in a specific way. It is not represented as funny or doubtful, neither is it suggested for consideration or inquisition. Rather the intuition “asserts” or “claims” the proposition to be true. In other words, an intuition represents its content as true (e.g. Bengson, 2015; Chudnoff, 2013; Huemer, 2001).^18^ This is how intuitions are TRUTH-ASSERTIVE.^19^ Intuitions appear to have this truth-assertiveness in common with beliefs and perceptual experiences. And it stands in contrast to e.g. imaginings which represent their content without making a claim about its truth or desires that insist on the fulfilment rather than the truth of their content.

Another aspect of intuitions is their PUSHY character.^20^ It is not only that they describe or “say” something (i.e. represent) and do it in a certain way (i.e. as true). At the same time, they also direct one to behave or act in a specific way towards their content, i.e. towards what they represent as true: they singlehandedly push or incline you to assent^21^ to their content, without e.g. additional desires (Chudnoff, 2013; Koksvik, 2021; Sosa, 2007; Van Inwagen, 1997).^22^

In contrast to a belief or judgment, however, an intuition does not fully *commit* the subject to its content. Instead, intuitions are “the tendencies that make certain beliefs attractive to us, that “move” us in the direction of accepting certain propositions without taking us all the way to acceptance” (Van Inwagen, 1997, p. 309). Insofar intuitions are NON-COMMITTAL.^23^ To illustrate consider:

For all conditions there is a set containing all and only the things meeting this condition. (Naïve Comprehension Axiom [NCA])^24^

If, after some reflection, *NCA* seems true to you then you are in good company. Many philosophers report that it seems true to them even though they have been convinced by Russell’s paradox that *NCA* is false (Bealer, 1992; Sosa, 2007; Williamson, 2007). Some philosophers make sense of this kind of “cognitive illusions” in analogy with optical illusions such as the Müller-Lyer lines (Bealer, 1992).

Another feature of intuitions is their GRADEABILITY. In fact, there are two aspects in which intuitions are said to be gradable. Call the first CONTENT-GRADEABILITY. Content gradeability concerns the way an intuition represents its content.^25^ It builds on a perceptual analogy: some perceptual experiences are more *determinate* or *vivid*, i.e. clear and distinct in representing their contents than others. Fitting glasses, for instance, make the content delivered by visual experience more determinate. Now, there (sometimes) seem to be a parallel quality to the content of intuitions, i.e. things that we see with the “mind’s eye” or hear with the “mind’s ear” can be more or less clear and distinct (Bengson, 2015; Chudnoff, 2013). On the other hand, there is PUSHINESS-GRADEABILITY (Koksvik, 2021; Weinberg, 2007). Naturally, we can be more or less strongly pushed towards assent (or dissent).

A peculiar feature of intuitions that sets them apart from states such as perceptual experiences, beliefs or imaginings is their PHENOMENAL EPISTEMIC VALENCE (Koksvik, 2021). So far we have considered *positive* intuitions, i.e. intuitions that represent some content as true. Now suppose it is 1963 and you believe that knowledge is justified true belief. You’re reading Edmund Gettier’s piece for the first time and arrive at Smith’s belief that *the man who will get the job has ten coins in his pocket* (Gettier, 1963). As it turns out, this belief is justified and true. Thus:

Smith knows that the man who will get the job has ten coins in his pocket. (Gettier)

Yet *Gettier* just seems false to you. Something similar might happen if you consider propositions such as:

2 + 2 = 5 (Calc Error)

There are as many even numbers as there are natural numbers. (Numbers)

Non-conscious physical duplicates of conscious beings are possible.^26^ (Zombies)

In such cases, what one experiences is a *negative* intuition where a proposition seems false instead of true.^27^ And this it does by *directly* representing a *specific* proposition as false. This stands in contrast to representing *Smith does not know that the man who will get the job has ten coins in his pocket* or *2* + *2* = *4* as true in response to *Gettier* and *Calc Error* or to having positive intuitions that represent a *nested* content such as *it is false that (Gettier)* or *(Calc Error)* as true.^28^ Crucially, positive intuitions differ *phenomenally* or in *how they feel* from negative intuitions (Koksvik, 2021). Interestingly, one can have a positive *or* a negative intuition about *Numbers* or *Zombies*. Note that in contrast to positive intuitions, negative intuitions will “assert” or “claim” a proposition to be false (rather than true) and push you towards dissent (rather than assent) concerning it.

Intuitions are NON-VOLUNTARY. The formation of an intuition is not an intentional act and not subject to direct voluntary control. One does not choose to have an intuition. While conscious choices, decisions, judgments, guesses or imaginings are sometimes under a certain degree of conscious control, intuitions are passively received—they happen or fail to happen to one (Bengson, 2015).^29^ As a consequence, one is considered less responsible or rationally criticisable for one’s intuitions than for one’s choices, decisions, judgments, guesses or imaginings (Chudnoff, 2013; Koksvik, 2021).

An upshot of these characteristics is that intuitions appear apt to play a perception-like JUSTIFICATORY role for judgments and beliefs: In virtue of just having an intuition that represents *Non-Contradiction* as true, you acquire some immediate justification to make the corresponding judgment, and while this justification is *prima facie* and defeasible, it does not require other justifying reasons. Thus, the way intuitions provide justification for corresponding intuitive judgments resembles the way perceptual experiences provide justification for corresponding perceptual judgments or beliefs (Bengson, 2015; Chudnoff, 2013; Koksvik, 2021).

## Issues with doxasticism and perceptualism

After having outlined these features, let’s take another look at currently available theories of intuitions. Consider Doxasticism: The most straightforward doxastic claim is that intuitions are beliefs or judgments. Contemplate for a moment the potential merits of this proposal. Doxasticism is parsimonious: it allows us to assimilate intuitions with familiar, canonical kinds of mental states—beliefs and judgments. A direct advantage of this assimilation is that we can make the knowledge that we have for beliefs and judgments bear on our understanding of intuitions, a state about which we initially know relatively little. This way we could advance from a description of the features of intuitions to an explanation of these features. If Doxasticism about intuitions is on the right track, then one might hope that the features of intuitions will fall out of the fact that intuitions are beliefs or judgments.

Can Doxasticism explain those features? Here we run into difficulties. If intuitions are beliefs or judgments, then it is puzzling that we appear less responsible or rationally criticisable for our intuitions than for our judgments or beliefs (Koksvik, 2021). It also does not seem that the pushiness of intuitions maps onto features of beliefs or judgments. An intuition singlehandedly pushes or inclines one to assent or dissent to its propositional content. In contrast, our assent to the contents of our beliefs lies in the past. Judgments, on the other hand, consist in the very mental act of assenting. In both cases, there is no pushiness reminiscent of intuitions. In fact, in the case of beliefs and judgments pushiness is not needed because, in contrast to intuitions, the question of whether to assent or not is already settled. This is just another way of saying that beliefs or judgments are essentially committal. Intuitions, on the other hand, are non-committal.

There are two more general challenges to the idea that intuitions are beliefs or judgments. Intuitions are characterized by having a specific phenomenology. In contrast, phenomenal features are usually not considered a characteristic element of beliefs and judgments (e.g. Klausen, 2013). Thus, the very idea that intuitions have a characteristic phenomenology conflicts with them being beliefs or judgments.^30^

Furthermore, intuitions are said to have justificatory powers similar to perceptual experiences: they can provide immediate justification for corresponding beliefs and judgments. In contrast, the justification conferred by beliefs and judgments is at best mediate: My belief or judgment that there is a rose in front of me or that there is exactly one line between any two points does not add to my justification for believing these things. The justification comes from and requires other sources such as perceptual experiences, testimony or intuitions.

In sum, we identify a significant amount of pressure points when trying to put the doxastic shoe on intuitions. In fact, Doxasticists often do not try to explain the phenomenology of intuitions in the first place. They rather tend to deny that there is something that needs explaining. They back this stance by reporting on their introspective inability to find such intuitive phenomenology among the contents of their conscious experience (see e.g. Williamson, 2007; Cappelen, 2012).

Now, I have mentioned earlier (see footnote 6) another common response to intuitions in the Doxastic camp. Take Williamson’s introspective report:

I am aware of no intellectual seeming beyond my *conscious inclination to believe* Naïve Comprehension, which I resist because I know better. I can *feel* such an inclination even if it is quite stably overridden (Williamson, 2007, p. 217, my emphasis).

Williamson admits that he feels something when having an intuition, namely a “conscious inclination to believe” (which is non-committal). To me this passage suggests that Doxasticists such as Williamson aren’t so much sceptical about intuitive phenomenology per se but about the *sui generis* states (e.g. “intellectual seemings”) often posited to make sense of it.

Now, what does a phenomenology-friendly Doxasticism offer as an alternative to make sense of such intuitions? It offers conscious inclinations to believe (or judge) (see also e.g. Sosa, 2007, 2014; Earlenbaugh & Molyneux, 2009; Cappelen, 2012). But now, what are these inclinations?^31^ One might perhaps have a “feel” for what is meant.^32^ This should not conceal the fact, however, that they aren’t some canonical kind of mental state such as beliefs or perceptual experiences.^33^ Against this background, Doxasticists (and Perceptualists, too) are too quick to let these inclinations pass as “familiar” and “doxastic” states. We just as much lack a satisfying account for these inclinations as for intuitions. Thus, inclinations themselves need to be explained just as much as the intuitions they are supposed to explain.^34^ For simplicity, when talking of “Doxasticism” from now on, I will have the phenomenology-*unfriendly* version in mind.^35^

Let’s now have a look at Perceptualism. This account takes the phenomenology of intuitions as its starting point. Perceptualists delineate intuitions phenomenally and use a perceptual analogy to zero in on some of the features of intuitions. The perceptual analogy not only allows to acknowledge that intuitions have a phenomenology but also that intuitions have specific properties (e.g. being non-committal) and roles (e.g. as non-inferential justifiers).

Now, can Perceptualism explain the features of intuitions? Looking at the first version of Doxasticism, we have seen how this can work: by identifying intuitions to be a mental state about which we know more than about intuitions themselves. This would allow us to tap into explanatory resources concerning the features of intuitions. A natural thought is: The perceptual analogy helps us to describe the features of intuitions—perhaps it can also explain them? Analogies by themselves, however, are of little explanatory value. They can become explanatory if they tap into a substantial relationship such as identity. This, however, appears outside the remit of Perceptualism. Perceptualists do not go as far as to say that intuitions *are* perceptual experiences—only that they are similar to them upon comparison.

If intuitions are neither beliefs or judgments nor perceptual experiences, what are they? According to Perceptualism, intuitions are *sui generis* states—a kind of mental state of their own. As a result, Perceptualism can acknowledge the properties and roles of intuitions but in doing so violate an important principle: parsimony. It is in this context that e.g. Michael Lynch reminds us of “a good commandment to live by, philosophically speaking. Namely, *thou shalt not posit mysterious faculties without necessity*” (Lynch, 2006, p. 231).

A significant pitfall with this, is that Perceptualism leaves us in a difficult place when it comes to explaining why intuitions have their features. This is because, in contrast to the knowledge we have about doxastic states and perceptual experiences, we do not have much independent knowledge about the postulated *sui generis* kind. In other words, the features of intuitions do fall out of assimilating them with the *sui generis* kind, but not in a particularly informative way. Perceptualism acknowledges and describes the features of intuitions but it does not make much progress in explaining them.

In sum, opting for Doxasticism seems a robust ontological option that, by way of reduction, promises explanatory gain. However, this ontological reduction does not fit well with the presented picture of intuitions and significantly reduces the scope of intuitive phenomenology and justification. Opting for Perceptualism does fit well but requires us to follow an uncertain ontological path and offers little explanatory gain.

## Looking at emotional experiences

At this point one might wonder: Are Doxasticism and Perceptualism the only ways to go? It seems premature to stop searching our mental ontology for a home for intuitions after having considered doxastic states and perceptual experiences only.

Affectivism comes into view if we don’t stop there. Here is a consideration leading up to it: intuitions are phenomenal states. This is why a Doxasticist analysis seems unsatisfying: beliefs and judgments are not paradigmatically phenomenal. This is also why a Perceptualist analysis is initially appealing: perceptual experiences are phenomenal states—in fact, they are *canonical* phenomenal states. So an idea is to look for other kinds of states that are canonical phenomenal states. At least one other mental kind naturally comes to mind: emotional experiences.

What are emotional experiences? A first extensional stab is to point towards experiences such as fears, joys and hopes. What do intuitions have in common with fears and hopes? More than is apparent on first sight. To see this, we need to identify some of the crucial commonalities that unite various emotional experiences into one species. These commonalities are found in the specific phenomenal and intentional features of emotional experiences.

First off, emotional experiences are phenomenally conscious, there is something “it is like” to have an emotional experience. Feeling happy about one’s meal or sad about one’s energy levels are phenomenally conscious states. But so are seeing blue and feeling air on one’s skin. However, only the former two are emotional experiences. So what distinguishes emotional from non-emotional experiences? That is, apart from being conscious, what are the marks of emotional experiences?

Arguably, the central feature of emotional experiences is PHENOMENAL VALENCE, i.e. the felt positivity or negativity of certain experiences (Charland, 2005; Teroni, 2018). This basic positivity or negativity is often conceptualised in hedonic terms as pleasantness or unpleasantness or in value terms as seeming value or disvalue (Carruthers, 2017a). Emotional experiences are essentially valenced experiences. Neither the visual experience of something blue nor the bodily sensation of air on one’s skin are felt as positive or negative by themselves. However, exteroceptive experiences and non-affective bodily sensations naturally prompt or co-occur with emotional experiences (such as sadness, enjoyment or fear) which do feel positive or negative. When I talk of valence, I mean valence as a phenomenal property of emotional experiences. Such phenomenal valence needs to be distinguished from associated but ultimately non-phenomenal properties such as emotion- or object valence (Colombetti, 2005).^36^

Valence, in turn, is closely associated with the ability of emotional experiences to directly motivate behaviour and action (Carruthers, 2017a; Corns, 2014). In other words, emotional experiences are MOTIVATIONAL: they exert a motivational force that can engender more or less specific behaviours. Furthermore, an emotional experience can motivate more or less strongly. In other words, the motivational force exerted by an emotional experience is GRADABLE (Kozuch, 2018).

Emotional experiences are plausibly assumed to be INTENTIONAL states (see e.g. Goldie, 2002; Tye, 2008; Kriegel, 2014). My fear of an approaching bear represents the bear as dangerous, your pride when being offered the coveted job represents the offer as an achievement. You might also be sad *that* your memory is degrading, or relieved *that* the number of new SARS-CoV-2 infections is going down. That is, emotional experiences represent that their intentional object (often called their *particular object*, be it a body part, an entity in the external world or a proposition) is a certain way. Namely that it bears some emotion-specific property such as being dangerous, a success, loss or improvement. These properties are called the formal objects of emotional experiences (Kenny, 1963; Teroni, 2007).^37^ In sum, the intentionality of emotional experiences has two parts: a particular object and a formal object where the emotion represents the former as bearing the latter.

In fact, in representing their particular objects as having an emotion-specific property, emotional experiences are ASSERTIVE in a specific way: they are assertive in that they “claim” that their particular object exhibits the emotion-specific formal object. The emotional experience in which you are afraid of a bear leaves little doubt that the bear *is* dangerous. In other words, emotional experiences assertively mark their particular objects as bearers of properties that are their formal objects—this can be danger, funniness or other properties for which we have dedicated emotional states. Fear is assertive about the presence of danger while amusement is assertive about the presence of funniness. In Bennett Helm’s words “emotions are […] a distinctive kind of passive assent to their targets as having the import defined by their formal objects” (Helm, 2001, p. 59).

Notwithstanding the fact that emotional experiences are potent engines of persuasion, they do not amount to full commitment to what they would want us to believe or judge. As everybody with phobias, jealousy or just less than optimal emotional responses would attest, emotions occasionally stand in conflict with our (better) judgment. One might be afraid of flying but, knowing the facts, not commit to the insisting suggestion of one’s fear—one believes and judges otherwise. Insofar emotional experiences are NON-COMMITTAL.

By the same token, we do not choose our emotions but are rather patients in respect to them, i.e. they are NON-VOLUNTARY. Michael Brady nicely summarises some of the mentioned aspects:

It is clear that emotional experience is typically passive: [...] [W]hen we feel an emotion the import of our situation impresses or thrusts itself upon us. [...] [T]o say that the import of a situation impresses itself upon S is to say, roughly, that S is inclined to assent to or endorse this view of the situation. In other words, when S experiences an emotion she is subject to some kind of pressure to accept the relevant appearance (Brady, 2009, pp. 420 sq.).

A characteristic feature of the intentionality of emotional experiences is that they rely for parts of their intentionality on other mental states such as bodily sensations, perceptions, judgments, memories, imaginings etc. These mental states provide emotional experiences with their particular object. Such a mental state is usefully called the *base* of an emotion (Deonna & Teroni, 2012; Mulligan, 1998). Emotional experiences are flexible in that they can take different kinds of states (or sets of those) with different kinds of contents (e.g. propositional/non-propositional, iconic/non-iconic, conscious/unconscious) as their bases. For instance, if one is afraid of an approaching bear, one’s fear relies on the multi-modal perceptual experiences of the bear in order to represent *the bear* as dangerous.

Against this background, emotional experiences are CONTENT-GRADABLE in an interesting way. First: they come with other mental states as their bases. Some of these bases such as perceptual experiences and imaginings will have content-gradeability, lending this feature to the emotional experiences that take them as their base. But there is more to the story: It is not only that the content of the emotion that is provided by the base will appear more or less vivid because the base has the feature of content-gradeablity. Crucially, the fact that it is the content of an emotion will make a characteristic *additional* contribution to the vividness of the content. This is suggested by ample empirical evidence that emotions facilitate content encoding in virtue of their affective properties such as valence (e.g. Kensinger & Corkin, 2004; Phelps et al., 2006; Schupp et al., 2003). That is, if paired with an emotional experience, representational content appears as more *vivid*.

A dominant view of the epistemology of emotions is conceiving the JUSTIFICATORY force of emotional experiences in analogy with perceptual experiences (e.g. Döring, 2009; Mitchell, 2017; Poellner, 2016).^38^ My fear of an approaching bear seems to immediately justify my judgment that the bear is dangerous in a similar fashion as my perceptual experience of the approaching bear immediately justifies my judgment that there is a tall, furry figure moving towards me. In both cases, this justification does not require other justifying reasons. At the same time, it is *prima facie* and defeasible—I might, for instance, also be aware of defeaters, say, that I am sitting in the living room of my downtown appartement and just ingested a hallucinogenic drug known to elicit disturbing experiences.

## Intuitions as emotional experiences

Recall that intuitions are (1) intentional, (2) truth/falsity-assertive, (3) pushy, (4) non-committal, (5) gradable in (5.1) content and (5.2) pushiness, (6) phenomenally epistemically valenced, (7) non-voluntary and (8) have a perception-like justificatory force. The central claim of Affectivism is that intuitions are emotional experiences. Affectivism should predict that intuitions have these features. Specifically, that intuitions have these features should follow from them being emotional experiences. Is this the case?

As we have seen, emotional experiences are INTENTIONAL. Thus, Affectivism predicts that intuitions are intentional. We find this to be the case. Now, one might want to make a restriction concerning the intentionality of intuitions, namely that they are specifically about propositions. Can emotional experiences be about propositions? On the face of it, they can: as mentioned above, emotional experiences are a class of mental states with a rich intentionality because they take their particular objects from the contents of other mental states that are their bases. These contents can supply particular objects for emotional experiences in the form of individuals but also propositions, such as when the base is a propositional imagining.^39^ With propositions as particular objects, intuitions would be no different from other emotional experiences that tend to be about propositions such as doubt, hope or despair (Deonna & Teroni, 2015; Wringe, 2015). Thus, Affectivism can also account for intuitions being about propositions.

By the same token, Affectivism has a story to tell about the CONTENT-GRADEABILITY of intuitions. Some mental states such as perceptual experiences or imaginings have native content-gradeability: they represent their contents more or less clearly, distinctly or vividly. Emotional experiences, in turn, take these (and other) kinds of mental states as their bases. In the case when an emotional experience takes a content-gradable mental state as its base, it inherits this feature since its particular object is represented more or less clearly, vividly or distinctly by the base. As a result, Affectivism predicts that intuitions, given a content-gradable base, represent their particular objects, typically propositions, more or less clearly, vividly or distinctly. Furthermore, the fact that the content becomes part of an emotional experience has an additional enhancing effect on how clearly, vividly or distinctly the content is represented. The visual representation of a bear is, *ceteris paribus*, more vivid if it is accompanied by fear than if it is not. As a consequence, Affectivism predicts that there will be a difference between the vividness of, say, just propositionally imagining *De Morgan’s Law* and propositionally imagining *De Morgan’s Law* accompanied by an intuition.^40^*Ceteris paribus*, *De Morgan’s Law* will appear more clearly, vividly or distinctly in the latter case.

Note that this picture does not exclude that *some* intuitions will not have content-gradeability. This is because some intuitions might have states as their bases that do not have native content-gradeability, say, certain kinds of thoughts.^41^ As a consequence, intuitions are unable to work their vividness-enhancing effect because there is no content that admits of being (made) more or less vivid. That there are intuitions with and without content-gradeability might partly explain why some prefer to speak of intuitions as perception-like experiences (e.g. Bengson, 2015) and others as inclinations (e.g. Sosa, 2007). It might be simply that the former have cases in mind where content-gradeability is more salient while the latter have cases in mind where the aspect of content-gradeability is less salient.

Affectivism correctly predicts that intuitions are NON-COMMITTAL and NON-VOLUNTARY. Note here a subtle contrast with what a full-blown perceptual analogy would predict: “Cognitive illusions” as in the case of *NCA* or *Numbers* are prime evidence for intuitions being non-committal and non-voluntary. At the same time, these cognitive illusions are usually made sense of in analogy with perceptual illusions such as the Müller-Lyer illusion. However, this analogy has its limits and should not be taken to suggest that one cannot rid oneself of one’s intuitions. As Christian Nimtz points out:

[O]ptical illusions are peculiar in that changes in our beliefs do not affect our perceptual experiences. Everyone will continue to have the impression of the lines differing in length in the Müller-Lyer illusion, whatever her knowledge of their actual length. But this does not hold true in the cases of intuitions [...]. Russell’s proof [...] might well make you *lose* the intuitions you used to have. (Nimtz, 2010, p. 366)

Thus, intuitions (that might lead to cognitive illusions) can be lost with sufficient efforts over which one does have voluntary control, say plentiful exposure to and recapitulation of counterevidence. This is a dissimilarity in comparison to optical illusions that seem to prevail no matter what.^42^^,^ 43 Thus, the perceptual analogy seems to overstate the degree to which we are at the mercy of intuitions’ non-voluntariness.

Now, Affectivism yields a slightly different prediction: if we look beyond the very moment of an emotion’s occurrence, we find ourselves capable of emotional learning in the sense that, given the right circumstances, practical knowledge and behavioural engagement, we can change the way emotional experiences make things appear. As a result, we can rid ourselves of illusory emotional experiences which represent things as being a certain way without them being that way. “Affective illusions” such as those brought about by phobias might lessen in strength and eventually dissipate. In other words, over time, you can change the way things feel. So the prediction yielded by Affectivism, in contrast to Perceptualism, gets things right. If intuitions are emotional experiences, then we should be able to change them over time (although typically not right away). This is what we find. Ultimately, cognitive illusions are not like perceptual illusions but like affective illusions: with time, effort and some know-how, we can rid ourselves of (illusory) intuitions—just as we can rid ourselves of (illusory) emotional experiences.

Intuitions are PUSHY: they singlehandedly push or incline you to assent or dissent to their content. Is this something Affectivism predicts? Remember, emotional experiences are motivational: they exert a motivational force that can engender all kinds of behaviours. An equally valid way of describing this is to say that they push or incline one to do something. Now, specific emotional experiences typically motivate specific behaviours that are in line with the way they make things appear (e.g. Döring, 2003). The motivational force that individual emotional experiences carry usually targets a specific range of emotion-congruent behaviours. The fear that represents an approaching bear as dangerous motivates one to escape (among other things). The anger that represents the remark of a neighbour as offensive motivates one to retaliate (among other things). What is important in the present context: emotional experiences not only motivate physical but also *mental* behaviour, such as attending, remembering and reflecting (e.g. Frijda, 2008). Now, there is no reason to exclude the mental behaviours of assenting or dissenting to a proposition from those that emotional experiences can motivate. Consequently, being pushy can simply be seen as a special case of being motivational: it is being motivational in respect to a specific mental behaviour: assent or dissent. Thus, Affectivism can account for the pushiness of intuitions. At the same time, we have seen that emotional experiences are gradable in the strength of the motivational force they carry. This predicts that the strength with which an intuition pushes one to assent or dissent will be gradable. In other words, Affectivism correctly predicts the PUSHINESS-GRADEABILITY of intuitions.

Let’s turn to the PHENOMENAL EPISTEMIC VALENCE of intuitions. The central datum about phenomenal epistemic valence is the observation that there are positive intuitions that directly represent propositions as true and that there are negative intuitions that directly represent propositions as false. Crucially, there is a marked difference in how positive intuitions *feel* compared to negative intuitions.

Note that, against the background of Doxasticism and Perceptualism, it is rather hard to make sense of phenomenal epistemic valence: Perceptual experiences do not seem to directly represent anything as false and thus appear to lack epistemic valence (or at least the negative side of it) and while beliefs might be thought to have an epistemic valence, this valence does not seem to be phenomenal (Koksvik, 2021; Martin & Dokic, 2013).^44^ It is no surprise, then, that in the absence of anything resembling phenomenal epistemic valence, Doxasticism is sceptical and Perceptualism inflationary about intuitions.

Now, Affectivism has resources to make sense of phenomenal epistemic valence: I have noted that the central feature of emotional experiences is their phenomenal valence. It is a general phenomenal property specific to and shared among emotional experiences (Weiss, 2016). Phenomenal valence makes experiences affective in the first place and marks some emotional experiences as positive and others as negative. Hope and amusement feel somehow positive in comparison to fear and embarrassment that, in turn, feel somehow negative. At times, in fact, some negative emotional experiences such as sadness and shame appear as phenomenal polar opposites of some positive experiences such as joy and pride (Mulligan, 2007). As far as I can tell, only the class of emotional experiences harbours such phenomenal polar opposites. This datum is naturally explained by the phenomenal valence of emotional experiences.

When it comes to positive and negative intuitions, now, Affectivism can proceed straightforwardly: positive intuitions feel different from negative intuitions because the former feel somehow positive in comparison to the latter that feel somehow negative. In other words, positive intuitions are positive emotional experiences and negative intuitions are negative emotional experiences. In fact, positive and negative intuitions naturally emerge as a pair of phenomenal polar opposites. The idea of Affectivism is, thus, that phenomenal valence is at the core of phenomenal epistemic valence.

However, there is still more to epistemic phenomenal valence than mere phenomenal valence. I said earlier that, for instance, amusement feels *somehow* positive and fear feels *somehow* negative. This is, emotional experiences usually do not feel positive or negative *simpliciter*. They feel positive or negative in a more specific way, namely, in relation to a significant property that is the formal object of the emotion. Amusement feels positive in relation to funniness while fear feels negative in relation to danger. Now, phenomenal epistemic valence does not only consist in the fact that positive intuitions feel different from negative intuitions but that the former is related to truth while the latter is related to falsity. Affectivism postulates the following in response: positive intuitions feel positive in relation to truth and negative intuitions feel negative in relation to falsity.^45^ In other words, positive intuitions have truth as their formal object while negative intuitions have falsity as their formal object. As a result, phenomenal epistemic valence emerges as a combination of phenomenal valence together with truth and falsity as formal objects. On the one hand, phenomenal valence is what makes intuitions emotional experiences, distinguishing them from non-emotional states. On the other hand, truth and falsity as formal objects is what makes positive and negative intuitions the *specific* emotional experiences they are, distinguishing them from other emotional experiences such as hope or despair.

With this realisation we have also arrived at the last feature of intuitions: TRUTH/FALSITY-ASSERTIVENESS. I said earlier that emotional experiences are assertive in relation to whether their particular object exhibits the property that is their formal object. Your fear upon spotting a bear does not merely suggest to you that the bear is dangerous. Rather it “asserts” that the bear *is* dangerous. Transposing this logic to intuitions, Affectivism predicts that truth/falsity-assertiveness is a special case of the general property of emotions being assertive. Intuitions are no different from other emotional experiences in “asserting” that their particular objects, propositions, exhibit the property that is their formal object, namely being true or false. This naturally complements the pushiness of intuitions: just as fear motivates us to escape *because* it represents an approaching bear as dangerous, intuitions push or incline us to assent or dissent to a proposition *because* they represent this proposition as true or false.

It now becomes clear how intuitions as emotional experiences can provide immediate justification for corresponding judgments and beliefs. If my intuition assertively represents a proposition as true or false, just as my amusement or my fear assertively represents a dog as funny or dangerous, then I seem to acquire some defeasible justification for a corresponding judgment. At the same time, the justification so acquired does not require other justifying reasons. As such, Affectivism fashions a perception-like JUSTIFICATORY force for intuitions.^46^

We can now see that all the features of intuitions fall out of Affectivism, giving it an explanatory advantage over Doxasticism and Perceptualism. These features can be made sense of as special cases of more general properties of emotional experiences. This is not surprising: although emotional experiences share some properties that unite them into one kind (e.g. having a phenomenal valence and a formal object), they express these properties in particular ways (e.g. having a specific phenomenal valence and formal object) that make them the specific emotional experiences they are.^47^

## Intuitions as epistemic feelings

Affectivism can acknowledge the features of intuitions. Furthermore, the fact that intuitions have these features is explained by them being emotional experiences. In fact, Affectivism effectively advances our understanding of the features of intuitions by applying the theoretical and empirical resources that we have for emotional experiences.

One might still be sceptical about the idea that intuitions are emotional experiences. It might not seem obvious upon introspection that intuitions are emotional experiences. This stands in stark contrast to the introspectable qualities of emotional experiences such as fears or joys. Intuitions are not obviously experienced as positive or negative (i.e. as emotional).

In this context, let me note on what Affectivism does *not* say: First, Affectivism does not say that it must be obvious that intuitions are emotional experiences. It is a surprising result that the feature profile of intuitions fits well with the features of emotional experiences, once one has broken them down and taken a closer look.

Now, one reason why one might be sceptical about Affectivism is because one thinks about emotional experiences in terms of “paradigmatic” emotional experiences, having their intense “violent” varieties in mind. In these cases it *is* introspectively obvious that one deals with emotional experiences. Such violent and obvious emotional exemplars are a rather rare occurrence, however. A much larger part of our emotional life is plausibly composed by the little, subtle movements of our emotional sensibilities. This becomes clearer if one considers that the function of emotional experiences is not to be violent but to make significant properties salient and prepare us to adaptively respond to them (Brady, 2009; Kozuch, 2018). Consequently, they typically direct our attention towards something else than themselves, towards something that matters. It is thus not surprising that we are only able to get a good look at them in exceptional circumstances—such as when they *are* violent and intense.

In fact, thinking emotional experiences back from their function provides further reason to believe that intuitions are emotional experiences. Consider the following question: Are truth and falsity (evolutionary) significant properties? In a tradition that at least dates back to Plato, we quite often talk of the value of truth and the disvalue of falsehood. It also seems that the properties of truth and falsity are of relatively high survival value to our species—a species that strongly relies on social coordination and the exchange of information. Furthermore, the significance of truth and falsity—in contrast to e.g. specific colours—is relatively invariant across contexts. These considerations, I contend, make it eminently plausible that we have evolved emotional experiences that swiftly detect truth and falsity in our external and internal milieus (Richter, 2015; Sperber et al., 2010).^48^

This brings us to a second point: Emotional experiences are a rich and varied class of mental states.^49^ Against this background, Affectivism only says that intuitions are emotional experiences. It does not (yet) say what kind of emotional experience intuitions are exactly. Intuitions are not best located among typically intense emotional experiences. I think that intuitions belong to a subclass of emotional experiences that is recently gaining in popularity: epistemic feelings.^50^ Epistemic feelings have been broadly described as “feelings that enter into the epistemic processes of inquiry, knowledge and metacognition” (de Sousa, 2008, p. 189). Here are some examples of epistemic feelings:

- FEELING OF FAMILIARITY: The feeling that some content is familiar (Whittlesea & Williams, 1998).
- FEELING OF KNOWING: The feeling that you are in possession of some relevant information (Koriat, 2000).
- TIP-OF-THE-TONGUE EXPERIENCE: The feeling that you are in possession of some relevant information but are unable to retrieve it (Schwartz & Metcalfe, 2011).
- FEELING OF (NOT) UNDERSTANDING: The feeling that you have (not) understood a certain content (Bowden et al., 2005; Dodd, 2014).
- FEELING OF COHERENCE: The feeling that some content is coherent or stands in relation to another content (Topolinski & Strack, 2009).
- FEELING OF RIGHTNESS/WRONGNESS: The feeling that some content is right/wrong (Mangan, 2001; Thompson et al., 2011).

There is good evidence that epistemic feelings are emotional experiences (Loev, 2022).^51^ However, epistemic feelings are different from their violent conspecifics in that they are a mild form of affect that makes us receptive to epistemic properties, comprises few bodily symptoms and notably motivates epistemic and mental behaviour. Due to their mild affective nature, they are not that obviously emotional and their emotional nature often tends to elude us. But this is no different from milder emotions in general which, plausibly, make up most of our emotional mental life (Schwitzgebel, 2008).

The class of epistemic feelings reminds us that there are mild forms of affect and shows that there is a suite of emotional experiences dedicated to epistemic properties. There are emotional experiences that detect familiarity, knowledge, coherence, rightness etc. Intuitions perfectly fit into this line. If intuitions are emotional experiences, then they are most plausibly epistemic feelings that usually come in the form of mild affect.^52^

Affectivism about intuitions might now seem like a plausible position. But is Affectivism true? Whether Affectivism is true is an empirical matter—for that we need to test its empirical predictions. Affectivism, for instance, predicts that we should be able to (at least sometimes) observe variations of affective markers in contexts where we judge something to be true or false.

Although we do not have conclusive empirical findings yet, there is already some preliminary empirical evidence for Affectivism worth mentioning: Experimental findings suggest that the (dis)fluency of cognitive processes associated with reading or hearing a statement (word recognition, memory retrieval, comprehension etc.) predicts whether one judges the statement to be true or false (see Dechêne et al., 2010 for a review). Processing (dis)fluency is the property of a specific process to be relatively fast (slow) (Reber et al., 2004). Thus, we know that processing fluency is linked to a heightened tendency to judge a statement as true. There are two other things that we know about processing fluency: it has been found to trigger positive affect and to proximally cause epistemic feelings (Alter & Oppenheimer, 2009; Winkielman et al., 2003). Thus, what we might observe in the mentioned experimental findings on truth judgments and processing fluency is that (fluency-triggered) positive affect guides truth judgments. Some researchers have begun to talk of “feelings of truth” in this context (Newman et al., 2012; Unkelbach et al., 2011). I suggest that what we are witnessing is, in fact, a specific kind of emotional experience and epistemic feeling at work: intuitions. In other words, we look at preliminary evidence for Affectivism. Further research should expand and substantiate this line of work.

## Conclusion

I have elaborated and made plausible Affectivism, the thesis that intuitions are emotional experiences. I further specified the thesis: specifically, intuitions are epistemic feelings, a subclass of emotional experiences that usually takes the form of mild affect.

Why is Affectivism preferable to Doxasticism and Perceptualism? Affectivism, like Perceptualism and unlike Doxasticism, acknowledges the substantial phenomenology and epistemology of intuitions. At the same time, it is, like Doxasticism but unlike Perceptualism, parsimonious when it comes to our mental ontology. Intuitions are states that are a well-established part of our mental ontology: emotional experiences.^53^

Certainly, the nature of emotional experiences themselves is not uncontroversial. What matters most, however, is that we have rich explanatory resources available for emotional experiences and that emotional experiences are themselves potent explanatory elements in a wide variety of psychological contexts. Affectivism, thus, finds intuitions a stable and potent place in our psychological life.

## Footnotes

1 John Bengson uses this example (Bengson, 2015, p. 711).

2 Proponents of Doxasticism are e.g. David Lewis, Timothy Williamson, Kirk Ludwig, Michael Lynch, Herman Cappelen and many experimental philosophers (e.g. Jonathan Weinberg). Proponents of Perceptualism are e.g. George Bealer, Michael Huemer, Joel Pust, Elijah Chudnoff, Ole Koksvik and Bengson.

3 Bealer uses this example (Bealer 1992, p. 102).

4 Lynch uses this example (Lynch, 2006, p. 229).

5 Chudnoff uses a similar example (Chudnoff, 2013, p. 152).

6 Not everybody agrees that intuitions have a phenomenology. According to a popular way of framing the available positions, Perceptualists embrace the phenomenology while Doxasticists deny it (e.g. Chudnoff, 2013). On a closer read, however, many Doxasticists grant that there is a phenomenology to intuitions (e.g. Williamson, 2007; Sosa, 2007; Earlenbaugh & Molyneux, 2009; Cappelen, 2012, see also Climenhaga, 2018). Unlike Perceptualists, however, Doxasticists are uncomfortable with making sense of this phenomenology by positing *sui generis* states. If you are a Doxasticist who is willing to grant phenomenology to intuitions but unwilling to grant that they are *sui generis*, then the present attempt at spelling out the phenomenal thesis in a *reductionist* way might leave you more at ease. I will hark back on this point in Sect. 3.

7 Two notes: First, in using terms such as “seem” or “appear” to refer to the phenomenology of intuitions, I follow common practice in the literature (e.g. Bealer, 1992; Huemer, 2001; Sosa, 2007). However, I thereby do not commit to views that flesh out these locutions to make sense of intuitions (Byerly, 2012; Tucker, 2013). Later I will explain the phenomenology of intuitions so picked out without recourse to these terms. Second, I grant that there are occasions when we use the terms “seem”, “appear” or “intuition” without having (in mind) an intuition in my sense and as depicted in Sect. 2 (Bengson, 2014, see also footnote 11).

8 This is a rough sketch, I will elaborate further on this assessment in Sect. 3.

9 Again, this is a rough sketch, I will elaborate on this in Sect. 3.

10 I specify what I mean by emotional experiences in Sect. 4.

11 Note that the intuition theories discussed here are evaluated on their merits as accounts of intuitions in the sense outlined in Sect. 2. This operates against the background that “intuition” talk, i.e. our uses of the word “intuition” and its cognates, is certainly highly varied. Sometimes we use “intuition” talk without referring to mental states at all but e.g. to commonsensical propositions or linguistic devices used to hedge epistemic risk (e.g. Bealer, 1992; Bengson, 2014; Cappelen, 2012; Smith, 2000). In fact, looking outside of philosophy one finds psychologists noting something similar: “There are as many meanings for the term intuition as there are people using it” (Betsch, 2008, p. 3) or that “intuition” “has been given so many different meanings, some opposite to others, that it makes one wonder whether the term has any meaning at all” (Epstein, 2008, p. 23). Naturally, then, Perceptualism and Affectivism grant that sometimes, perhaps often, we mean doxastic states such as beliefs (that are perhaps commonsensical or unreasoned) or judgments (that are perhaps prompt) when talking about “intuitions”. The point of disagreement with Doxasticism is about whether there is more to the mental states we call “intuitions” than beliefs or judgments (and potentially other doxastic states). This disagreement is well compatible with “pluralism” about what the word “intuition” refers to, including, say, some doxastic states. Thus, Perceptualism and Affectivism reject Doxasticism only as an account of intuitions in the sense outlined in Sect. 2 and not as an account of some other things we call “intuitions”. At the same time, Affectivism accounts for the same data as Perceptualism (i.e. intuitions in the relevant sense) without positing *sui generis* states, rendering Perceptualism redundant. Thus, the question of whether Perceptualism accounts for some other things we call “intuitions” does not come up. I would like to thank an anonymous reviewer for suggesting this clarification.

12 I think that there are features among them that are found necessarily and exclusively in instances of the target state. To anticipate: the most promising candidates for these features are truth/falsity-assertiveness and phenomenal epistemic valence. These features are distinctive of intuitions. Nevertheless, sticking only to these features would conceal much of their complexity.

13 Bealer uses this example (Bealer 1992, p. 101).

14 Koksvik uses this example (Koksvik, 2021, p. 25).

15 Chudnoff uses a similar example (Chudnoff, 2013, p. 152).

16 This is not to exclude that intuitions can be about non-propositional objects (see e.g. Bonjour, 2001, p. 676 sqq.; Chudnoff, 2013). However, if not indicated otherwise I will focus on propositions as the intentional objects of intuitions for now.

17 I will refer to the intentional object of an intuition in form of a proposition as the content of the intuition.

18 Or false, see further below.

19 Or FALSITY-ASSERTIVE, see further below.

20 I borrow the term “pushy” from Koksvik (2021).

21 Or dissent, see further below.

22 In the literature there is an ambiguity of whether pushiness is to be understood as a *felt* push or as just somehow establishing a *disposition* to do something without implying pushy phenomenology. I take this to be the difference between talk of intuitions as inclinations (e.g. Sosa, 2007) and dispositions (e.g. Sosa, 1996). The account to be developed here will focus on pushiness understood phenomenally. Insofar it can also be taken as an elaboration of the nature of intuitions as inclinations to believe or judge (see also Sect. 3).

23 This is only to say that having an intuition does not by itself amount to commitment to its content. For that we must *additionally* assent to it by judging accordingly. We typically but not always judge accordingly (see below).

24 Bealer uses this example (Bealer, 1992, p. 102).

25 Note that content-gradeability is a feature of the experience, not its content.

26 Bengson uses this example (Bengson, 2015, p. 712).

27 Being positive or negative is different from being correct or illusory. For instance, the *NCA* intuition is positive because it represents *NCA* as true but illusory because *NCA* is false.

28 That is not to say that if one has an intuition in response to propositions such as *Numbers* one *always* has directly corresponding negative intuitions. It is possible that one can just as well have positive contradicting intuitions (with nested contents).

29 Although one might actively work towards having or losing an intuition.

30 What about distinctive cognitive phenomenology? Proponents of cognitive phenomenology claim that besides the phenomenology of perceptual, somatic and affective states there is also a (distinctive) phenomenology to purely cognitive states such as beliefs and judgments (see e.g. Bayne & Montague, 2011). The idea of cognitive phenomenology is controversial and subscribing to it to account for intuitions is a significant theoretical commitment. I take it to be a virtue of Affectivism that it can spare us this commitment, relying on less controversial ingredients (i.e. emotional experiences). On any count, Doxasticists usually do not appeal to cognitive phenomenology to explain the phenomenology of intuitions. In fact, Doxasticists often deny that there is any phenomenology in need of explanation, cognitive or otherwise (see further below in the main text). It is thus a yet unexplored question whether the alleged cognitive phenomenology of beliefs can account for the phenomenology of intuitions. Even if one grants that intuitions have a phenomenology and even that beliefs have a cognitive phenomenology, it is still not clear whether the phenomenology of intuitions is the same as the cognitive phenomenology of beliefs, whether intuitions have a unique cognitive phenomenology of their own or whether the phenomenology of intuitions is a cognitive phenomenology at all. That an account of the phenomenology of intuitions using the alleged cognitive phenomenology of beliefs might not work is attested by the route usually taken by Perceptualists. Perceptualists often do subscribe to cognitive phenomenology and their accounts of intuitions are to be understood as built on this kind of phenomenology (e.g. Chudnoff, 2013; Koksvik 2021). However, they do not go on to make sense of intuitions as beliefs but as other kinds of *sui generis* states with a cognitive phenomenology. This highlights the fact that even if one would grant beliefs cognitive phenomenology, this belief-specific cognitive phenomenology would be ill-suited to account for intuitions in the relevant sense.

31 On some readings, these “inclinations” are just *dispositions to believe* (Sosa, 1996; see Audi, 1994 for the notion). Such a reading, however, seems to undermine the phenomenology and epistemology of intuitions. It is consensus that experiential “mental phenomena are necessarily occurrent. There are no dispositional experiential phenomena” (Strawson, 2009, p. 159). Furthermore, it is unclear how a disposition to believe a proposition has any bearing on the justification for believing this proposition.

32 Though this is hardly more than the “feel” one might have for what is meant by “intellectual seemings”.

33 The notion of “canonicity” here is supposed to capture that the existence and place of some mental states such as beliefs or perceptual experiences are less controversial than the ones of others—such as the mentioned inclinations or intellectual seemings.

34 If you are interested in the fate of said inclinations, stay tuned—I submit that the account of intuitions to be developed here can elucidate their nature just as well.

35 One might wonder: if the alleged claim that beliefs are essentially committal is a problem, could one conceive of an alternative doxastic account of intuitions that treats them as graded but not all-out beliefs? Although I am not aware of such an account of intuitions (in the relevant sense), such an account is certainly conceivable, and I am looking forward to a forthcoming argument for it. Let me lose a few words on its prospects. First, such an account can take many different forms. (1) One (as I have argued uninformative) way of conceiving of “graded beliefs” is just to point towards the already mentioned inclinations to believe or judge. (2) Another way is to conceive of them as (educated) guesses (Cohen, 1974; Dorst & Mandelkern, 2021), (3) in-between beliefs (Schwitzgebel, 2001), (4) credences (Jackson, 2020), or (5) in yet other ways (e.g. the ones reviewed by Bayne & Hattiangadi, 2013). Before a specific account is put forward, it is difficult to adjudicate between these accounts (with their many nuanced commitments and uncertain outcomes: are, for instance, credences beliefs or vice versa?) and would take us here too far afield. Second, I want to note again that I do not deny that we sometimes call doxastic states such as beliefs, judgments, or some kind of graded belief “intuitions” (see footnote 11). The point of contention is whether these would be the intuitions in the relevant sense outlined in Sect. 2. One might doubt this since even graded beliefs might be conceived as essentially committal attitudes (beliefs) with a probabilistically graded content, something like: If I believe that *likely p*, I still commit to the probabilistic proposition *likely p* and this is a problem as intuitions as attitudes appear to be non-committal. Equally there appears to be a problem if we conceive of graded beliefs as being a less than committal kind of attitude. This is because a graded belief would still assign some degree of trust (e.g. credence) to a proposition that is believed. However, in cases such as the *NCA* intuition, I do not assign any trust to *NCA*, I do not believe it *at all*, and yet I still have the intuition that it is true. Thus, no degree of trust seems to be assigned to the intuitive proposition, making it hard to reconcile with the graded-attitude-belief-view. Finally, while it might be easy to understand what it means to have some reasons for believing something—lending it some degree of support *for* actually believing it—epistemically speaking, it is unclear how this maps onto a psychological reality and whether and what kinds of states embody such facts in our experience. As phenomenological explorations of graded beliefs appear to be yet scarce, such an account of intuitions would have to cover a lot of new ground. I find it an exciting possibility that (a portion of) the psychological reality of such graded beliefs might, in fact, lie in the emotional experiences that I propose intuitions to be. It might well be that my graded belief in *p* expresses itself in my graded emotional experience, my intuition, that *p* (Cohen, 1989; Schwitzgebel, 2002). I would like to thank an anonymous reviewer for making me consider the prospects of graded beliefs in this context.

36 Object-valence and emotion-valence refer to whether a stimulus or emotion is negative (e.g. stimuli: angry or sad faces, snakes; emotions: fear, sadness) or positive (e.g. stimuli: happy and attractive faces, tasty food; emotions: joy, amusement) as such, regardless of whether the object elicits emotions or how the emotion feels.

37 I am naming the mentioned formal objects just as plausible candidates. Settling the question about the exact (distal) properties that specific emotional experiences are receptive to is an empirical project.

38 This kind of Epistemic Perceptualism about emotions is not uncontested (see e.g. Brady, 2013) but I cannot argue for it here and will assume it on the right track. (Metaphysical) Affectivism about intuitions does not entail Epistemic Perceptualism about intuitions but it does establish it as a plausible option. An advantage of bringing the discussion of Epistemic Perceptualism about emotions to bear on intuitions is its advanced state.

39 Here, the notion of propositional imagining where the content of an imagining is propositional (e.g. imagining that *De Morgan’s Law*) contrasts with objectual imaginings where the content of an imagining is objectual (e.g. imagining *De Morgan’s Law*) (Yablo, 1993). Apart from allowing to make this distinction, propositional imagining can be understood along minimalistic lines as captured by Kathleen Stock: Propositional imagining “involves, minimally, taking a certain attitude of ‘thinking that’ to a given content that one reads [or hears etc.], without a commitment to its truth, any automatic integration with one’s belief set, or any automatic relevance to one’s behaviour. It may not involve a substantial phenomenological aspect. It can be largely passive and involve little deliberate activity on part of the reader [or listener etc.] other than reading [or hearing etc.] and processing of lines of text [or heard utterances etc.]. (Stock, 2017, p. 27).

40 What is the difference between imagining that *De Morgan’s Law* and a *De Morgan’s Law* intuition occurring upon imagining that *De Morgan’s Law*? While the former case is characterized by neutrally entertaining the thought that *De Morgan’s Law*, the latter case is additionally characterised by the features outlined in Sect. 2, i.e. *De Morgan’s Law* is represented to be *actually* true and you are e.g. pushed to assent to it (see also main text for differences in content-gradeability).

41 I want to leave open which (propositional) states might lack native content-gradeability. There might, for instance, be propositional imaginings that are perception-like and those that are belief-like. I see no reason to deny content-gradeability regarding the former but one might want to deny content-gradeability regarding the latter.

42 At least if one has them in the first place (Henrich et al., 2010; Segall et al., 1966).

43 Perhaps the “no matter what”-formulation is too strong. However, to my knowledge there are no reported cases where subjects (without neurophysiological damage) could be made to lose the illusory Müller-Lyer experience.

44 Though those subscribing to cognitive phenomenology might disagree.

45 This idea does neither imply that truth and falsity are somehow *intrinsically* positive or negative nor that our *only* access to truth and falsity as properties lies in emotional experiences. The claim is rather that the properties of truth and falsity are sometimes represented by positive and negative emotional experiences—intuitions. We can judge something to be true without an associated intuition just as we can judge something to be dangerous without an associated fear experience. Furthermore, there is nothing that precludes a (strong) *negative* affective reaction to something that one initially takes to be true based on a (subtle) *positive* affective reaction, namely an intuition. The idea that the truth or falsity of some contents are sometimes conveyed by specific positive or negative emotional experiences does not by itself imply that the conveyed properties and contents are positive or negative independently of the specific emotional experience.

46 Spelling out the epistemic implications of Affectivism in detail must be left for another occasion.

47 One might wonder: Why believe that intuitions are emotional experiences and not merely associated with emotional experiences? First, a mere association claim brings us back to square one in accounting for intuitions and their features—for what are the intuitions then? Second, it is exactly the resources that emotional experiences bring to the table that allow us to account for the features of intuitions. This would be an odd coincidence if the relationship is merely associative.

48 This is not to say that our suite of truth- and falsity-detecting mechanisms is *exhausted* by these emotional experiences (see also footnote 46).

49 Nevertheless, this class is united by the properties outlined in Sect. 4.

50 Epistemic feelings go also by other names such as epistemic emotions or noetic feelings (e.g. Carruthers, 2017b; Dokic, 2012).

51 Epistemic feelings can be conceptualised as a subclass of emotions alongside other subclasses such as aesthetic emotions or social emotions (see e.g. Carruthers, 2017b).

52 Also Jennifer Nagel and Miguel Egler mention intuitions and epistemic feelings in one breath (Egler, 2020; Nagel, 2007). Their projects are distinct from mine. Nagel is a Doxasticist about intuitions and reviews the feeling of knowing as an antecedent of some epistemic intuitions. Egler notes on the similarities between the feeling of rightness and intuitions to make a metaphilosophical point. He does not aim to provide an account of intuitions or to establish them as emotional experiences.

53 Additionally, emotional experiences are not only canonical mental states but also canonical *phenomenal* states, i.e. mental states with a distinctive phenomenology. Identified as emotional experience, intuitions thus not only inherit the firm ontological grounding of a canonical mental state but their phenomenology can also be accounted for along the lines of a canonical phenomenology, namely emotional phenomenology. This way, one would, for instance, not need to subscribe to the existence of *sui generis* cognitive phenomenology (see also footnote 30).
